# A non-anhydrous, minimally basic protocol for the simplification of nucleophilic ^18^F-fluorination chemistry

**DOI:** 10.1038/s41598-020-61845-y

**Published:** 2020-04-22

**Authors:** J. A. H. Inkster, V. Akurathi, A. W. Sromek, Y. Chen, J. L. Neumeyer, A. B. Packard

**Affiliations:** 1 Division of Nuclear Medicine and Molecular Imaging, Boston Children’s Hospital, Boston, MA, 02115 USA; 2 Harvard Medical School, Boston, MA, 02115 USA; 3 Division of Basic Neuroscience, McLean Hospital, Belmont, MA, 02478 USA

**Keywords:** Synthetic chemistry methodology, Nuclear chemistry

## Abstract

Fluorine-18 radiolabeling typically includes several conserved steps including elution of the [^18^F]fluoride from an anion exchange cartridge with a basic solution of K_2_CO_3_ or KHCO_3_ and Kryptofix 2.2.2. in mixture of acetonitrile and water followed by rigorous azeotropic drying to remove the water. In this work we describe an alternative “non-anhydrous, minimally basic” (“NAMB”) technique that simplifies the process and avoids the basic conditions that can sometimes limit the scope and efficiency of [^18^F]fluoride incorporation chemistry. In this approach, [^18^F]F^−^ is eluted from small (10–12 mg) anion-exchange cartridges with solutions of tetraethylammonium bicarbonate, perchlorate or tosylate in polar aprotic solvents containing 10–50% water. After dilution with additional aprotic solvent, these solutions are used *directly* in nucleophilic aromatic and aliphatic ^18^F-fluorination reactions, obviating the need for azeotropic drying. Perchlorate and tosylate are minimally basic anions that are nevertheless suitable for removal of [^18^F]F^-^ from the anion-exchange cartridge. As proof-of-principle, “NAMB” chemistry was utilized for the synthesis of the dopamine D_2_/D_3_ antagonist [^18^F]fallypride.

## Introduction

**Figure 1:**
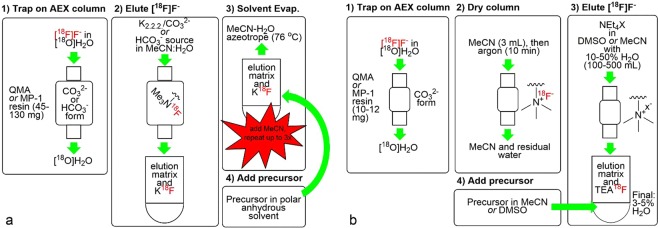
Traditional (**a**) and “non-anhydrous, minimally-basic” NAMB (**b**) approaches to the extraction, preparation and use of [^18^F]F^−^ for the manufacture of PET radiopharmaceuticals. X^−^ = HCO_3_^−^, ClO_4_^−^, or OTs^−^.

The production of ^18^F-labeled compounds for Positron Emission Tomography (PET) typically follows a set of conserved steps for the preparation of reactive [^18^F]F^−^ (Fig. 1a). The cyclotron-produced [^18^F]F^−^ is extracted from the [^18^O]H_2_O target using a quaternary methylammonium (QMA) or macroporous QMA (MP-1) anion-exchange (AEX) resin^1^. The [^18^F]F^−^ is then eluted from the resin using an MeCN-water solution of K_2_CO_3_ or KHCO_3_ and Kryptofix 2.2.2. (K_2.2.2_), with the CO_3_^2−^ or HCO_3_^−^ anion displacing the [^18^F]F^−^ from the AEX resin and K_2.2.2_ acting as a phase-transfer catalyst (PTC) in the subsequent ^18^F-fluorination reaction^2^. Alternative eluents include tetrabutylammonium bicarbonate (TBAB) and tetraethylammonium bicarbonate (TEAB), in which the tetra-butylammonium cation serves as a PTC. The water is then removed from the eluate by azeotropic distillations in which successive portions of acetonitrile are added to the vial and then removed by heating under a stream of inert gas. This “drydown” process is considered crucial to the success of ^18^F radiolabeling because hydrated fluoride is generally thought to be poorly nucleophilic^3^. After the [^18^F]F^−^ has been dried, a solution of the precursor compound in an anhydrous solvent is added to the dried [^18^F]F^−^, and the reaction mixture is heated to produce the desired product.

This technique is reliable and extraction of [^18^F]F^−^ from the AEX cartridges is typically very high. There are, however, limitations to this approach. These include: 1. the fact that many precursor compounds and ^18^F tracers do not tolerate basic reaction conditions very well, especially at high temperatures; 2. small variations in the drydown step are often cited as a factor in the batch-to-batch differences in yield observed during the production of ^18^F radiopharmaceuticals for clinical use; and 3. some [^18^F]F^−^ is always lost due to volatilization and adsorption of the dried K[^18^F]F onto the walls of the reaction vessel during the drydown process. Eliminating these limitations could improve the reliability of ^18^F radiopharmaceutical production.

There have, therefore, been multiple efforts to improve this process. These have included: (1) The use of alternative AEX sorbents, including an *N*-vinyl lactam/divinylbenzene copolymer preloaded with long-chain quaternary ammonium salts^4^ and C_18_ cartridges impregnated with a phosphonium borane^5^; (2) The use of elution matrices containing strong organic bases (e.g., phosphazenes^6^ and pyridines^7^), inert salts (e.g. potassium mesylate^8^, [bmim][OTf]^9^), or anhydrous solutions of [K_2.2.2._]OH^10^; and (3) The use of a TiO_2_ catalyst to carry out ^18^F-fluorinations without isolating the [^18^F]F^−^ from the target [^18^O]H_2_O^11^. Other examples include the work of Richarz, *et al*. who described a “minimalist” approach in which [^18^F]F^−^ is eluted from QMA cartridges using MeOH or EtOH solutions where the cation is the trimethylanilinium, diaryliodonium, or triarylsulfonium derivative of the aromatic target compound and the anion is TfO^−^ or Br^−^ ^12^. The cation serves as the PTC and the TfO^−^ or Br^−^ counterion displaces the [^18^F]F^−^ from the AEX resin. However, the eluting solvent must still be removed by distillation and replaced with a polar aprotic reaction solvent. Neumann, *et al*. described a related approach in which [^18^F]F^−^ is eluted from the AEX cartridge using uronium salt-based precursors in 10:1 2-butanone:EtOH, and the ^18^F-labeling reaction is carried out after addition of tributylamine to the eluate^13^. While not a general approach to [^18^F]F^−^ incorporation, this approach permits the incorporation of [^18^F]F^−^ into electron-rich (*i.e*. unactivated) aryl rings, and thus stands to make a significant impact in this research area. Another example of an innovative way to avoid the drydown step is the work of Basuli, *et al*. who discovered that certain ^18^F-fluorination reactions will take place on the surface of a QMA resin at room temperature. Using this method, they prepared the prosthetic group [^18^F]fluoronicotinic acid-2,3,5,6-tetrafluorophenyl ester by passing the trimethylammonium triflate precursor through an AEX cartridge impregnated with [^18^F]F^−^ ^14^. The ^18^F-labeled product was then immediately added to solutions containing amine-bearing targeting vectors [*e.g. c*(RGDfK), albumin, and a prostate-specific membrane antigen (PSMA) inhibitor] to produce the [^18^F]fluoronicotinic amides^15^.

The simplest approach to avoiding the drydown step is to use the [^18^F]F^−^ eluted from AEX cartridges directly, without azeotropic drying. In one example of this approach, Brichard and Aigbirhio^16^ used high concentrations of TEAB (15 mg/mL) in large elution volumes (1 mL) to elute [^18^F]F^−^ from 130 mg Sep-Pak® QMA cartridges. The decay corrected (DC) elution efficiencies using these “damp” solutions of MeCN or DMF were 95–99% and 88–99%, respectively. When using DMSO, 5% water was required in order to achieve an elution efficiency of 89%. Subsequently, 100 µL fractions of the eluate, each containing 7.8 µmol (1.5 mg) TEAB, were used for the synthesis of several clinically relevant PET tracers, including [^18^F]fallypride [maximum radiochemical conversion (RCC) = 58% by HPLC], [^18^F]fluoroethyltosylate (94% RCC) and 4-[^18^F]fluorobenzonitrile (79% RCC). [Note that in the context of this discussion, RCC refers to labeling efficiency, typically determined by TLC or HPLC analysis of the crude reaction mixture, not isolated radiochemical yield (RCY)^17^. This distinction is necessary because in many cases the investigators did not isolate the product from the reaction mixture.] Another example is the work of Blecha, *et al*., who reported the quantitative elution of [^18^F]F^−^ from a 12.6 mg MP-1 cartridge (ORTG, Inc.) using 1–2 mL solutions of K_2_CO_3_/K_2.2.2._ in 97% DMF or 99% MeCN^18^. Several aromatic and aliphatic substrates were successfully radiolabeled (e.g. 4-[^18^F]fluorobenzonitrile from 4-nitrobenzonitrile, 50% RCC; from 4-trimethylammonium triflate benzonitrile, 95% RCC) by mixing a fraction of the eluate (1/8 to 1/4 of the total radioactivity) with a solution of the precursor in dry solvent. It’s important to note that in both of these cases only a small fraction (10–25%) of the total amount of the [^18^F]F^−^ eluted from the AEX cartridge was used in each labeling reaction, which significantly reduces both the amount of base and the amount of water in the reaction mixture.

Kniess, *et al*. prepared [^18^F]fluoroethyltosylate using [^18^F]F^−^ in K_2.2.2._ and K_2_CO_3_, KOH, or K_2_C_2_O_4_ solutions containing 2–5% water in MeCN^19,20^. The [^18^F]F^−^ was directly eluted into a vial containing the ethylene di(*p*-toluenesulfonate) precursor (7 mg) and Cs_2_CO_3_ (6.5 mg)^21^ which was then heated (100 °C, 15 min) to produce [^18^F]fluoroethyltosylate (RCC = 76–96%). This synthesis used large amounts of a robust precursor (7 mg) and carbonate (50 µmol total), so additional studies are probably warranted to establish the substrate scope. Most recently, Kwon *et al*. reported 86% [^18^F]F^−^ elution efficiency from 46 mg QMA columns using a solution of K_2.2.2_/K_2_CO_3_ in 96 µL H_2_O in MeCN (600 µL total volume) water/MeCN mixtures. The eluate was then diluted with a solution of the precursor (1–3 mg) in 1.4 mL DMSO or DMA, and the resulting reaction mixtures were heated (140 °C, 10 min) to afford [^18^F]PSS232, [^18^F]MISO and [^18^F]fallypride^22^. The RCCs ranged from 15–46% (measured by HPLC). Notably, they observed that the RCC of [^18^F]fallypride prepared by manual synthesis (45%) was similar to that obtained using an automated synthesizer (46%), suggesting that a non-anhydrous approach at this scale might be amenable to the clinical production of ^18^F PET tracers. It’s worth noting that the RCY of [^18^F]fallypride was only 25%, slightly more than half the RCC, highlighting the distinction between RCC and RCY and the fact that RCCs measured by HPLC often overestimate [^18^F]F^−^ incorporation^23^.

The following report describes an approach that, informed by these previous studies, facilitates the synthesis of ^18^F-labeled PET tracers under non-anhydrous reaction conditions (3 or 5% water in 1 mL total solvent; Fig. 1b). In contrast to the approach of Brichard and Aigbirhio^16^, this approach uses the entire volume of [^18^F]F^−^ eluted from the anion exchange. The reaction conditions thus reflect typical radiopharmaceutical production conditions. This work also introduces the use of minimally basic tetraethylammonium salts (vs. HCO_3_^−^ or CO_3_^2−^) as [^18^F]F^−^ eluents with the expectation that the resulting [^18^F]F^−^ solutions might prove more suitable for ^18^F-labeling of base-sensitive precursors. Finally, we report a protocol for the synthesis of [^18^F]fallypride as the proof-of-concept for this “non-anhydrous, minimally basic” (NAMB) approach.

## Results and Discussion

Commercially available AEX columns containing 10–12 mg of standard QMA (capacity = ~0.2 meq/g) or MP-1 (capacity = ~0.7 meq/g) resin were used for [^18^F]F^−^ trapping. These small columns were connected to disposable syringes *via* a Luer-lock/hose barb adapter (Supplementary Fig. S2). After the [^18^F]F^−^ was trapped on the column, the column was washed with acetonitrile (3 mL) and argon was passed through the column for 10 min (Fig. 1b). The [^18^F]F^−^ was then eluted with a tetraethylammonium salt in 100–500 µL of MeCN or DMSO containing 10–50% water. We attempted to use lower water concentrations; however, at the lower water concentrations described by Brichard and Aigbirhio (0–2% H_2_O, 15 mg/mL TEAB)^16^, we observed that TEAB precipitated from the solution upon standing. Increasing the water content to 10–50% ensured that the salt remained in solution and facilitated [^18^F]F^−^ extraction. After elution, a solution of the precursor in anhydrous solvent was added, reducing the final water concentration to 3–5% in a 1 mL reaction volume.

Table 1 summarizes the optimization of the parameters for [^18^F]F^−^ elution from the MP-1 AEX columns. We first investigated TEAB (7.8 µmol, 1.5 mg) in 500 µL 90% MeCN or DMSO/10% H_2_O. In agreement with Brichard and Aigbirhio^16^, MeCN proved to be a superior eluent to DMSO. Decreasing the TEAB concentration by half (3.9 µmol in 500 µL of 90% MeCN/10% H_2_O) decreased the elution efficiency from 89% to 76% (entries 2 & 3), and replacing MeCN with DMSO decreased the elution efficiency from 89% to 59% (Table 1, entries 2 & 4). Considering that some ^18^F PET tracers suffer from low isolated yields due to the sensitivity of the precursor or product to heating in the presence of HCO_3_^−^ or CO_3_^2−^, we also evaluated tetraethylammonium perchlorate (TEAP) and tetraethylammonium *p*-toluenesulfonate (TEATos) as minimally basic alternatives to TEAB. In this case, the only base present in the final “NAMB” reaction mixture was the small amount of carbonate present on the column after pre-conditioning by the manufacturer that is co-eluted with the [^18^F]F^−^.

**Table 1:** Efficiency of [^18^F]F^−^ elution from MP-1 columns using tetraethylammonium salts in organic solvent-water mixtures.

Entry	AEX Reagent	Elution Volume (µL)	Elution Matrix	% Elution Efficiency (±σ)	*n*=
1	TEAB	500	100% H_2_O	97 (<1)	3
2	TEAB	500	90% MeCN	89 (6)	5
3*	TEAB	500	90% MeCN	76 (7)	4
4	TEAB	500	90% DMSO	59 (4)	3
5	TEAB	100	50% MeCN	95 (2)	3
6	TEAB	100	50% DMSO	92 (3)	5
7	TEAP	100	50% MeCN	94 (2)	5
8	TEAP	100	50% DMSO	94 (4)	10
9	TEATos	100	50% MeCN	95 (3)	3
10	TEATos	100	50% DMSO	95 (1)	2
11	TEAB	100	70% MeCN	93 (<1)	4
12	TEAB	100	70% DMSO	85 (4)	3
13	TEAP	100	70% MeCN	90 (6)	6
14	TEAP	100	70% DMSO	80 (4)	5
15	TEATos	100	70% MeCN	95 (2)	14
16**	TEATos	100	70% MeCN	95 (1)	16
17	TEATos	100	70% DMSO	89 (5)	3

Mass of MP-1 resin was 10–12 mg. Elution efficiencies are DC. The mass of the tetraethylammonium salt was 7.8 µmol unless noted otherwise. Owing to its explosive potential when dry, TEAP is typically sold ‘damp’ (~10% water), which hampers an accurate measurement of TEAP mass. *TEAB = 3.9 µmol. **QMA column used (carbonate form).

Columns were reversed before elution of the [^18^F]F^−^. Elution in the same direction as [^18^F]F^−^ capture resulted in lower elution efficiency (<60%) in all cases, except when eluting a QMA column using 7.8 μmol AEX reagent in 50% MeCN/water (100 μL). Under these conditions, the elution efficiencies in the forward direction using TEAB, TEAP, and TEATos were 82, 79, and 98% respectively.

Entries 5–10 in Table 1 summarize the efficiency of [^18^F]F^−^ elution using 7.8 µmol of TEAB, TEAP or TEATos in 100 µL of 50% organic solvent/50% water. In these examples, the elution efficiency was higher (92–95%) than with 500 µL of 90% organic solvent/10% water, despite the smaller elution volume. Furthermore, when the eluent contained 50% (vs. 10%) water, MeCN showed no advantage over DMSO. Although beneficial during elution of [^18^F]F^−^ from the column, the additional water may have a deleterious effect on subsequent ^18^F-fluorination chemistry, so we also evaluated eluents containing 30% water (entries 11–17). Under these conditions, TEAP in 70% MeCN (90% elution efficiency) was slightly inferior to TEAB (94% elution efficiency) and TEATos (94% elution efficiency). As expected, [^18^F]F^−^ elution efficiency was higher with 70% MeCN than with 70% DMSO. When TEATos in 70% MeCN was used as eluent, there was no apparent difference in elution efficiency between the QMA and MP-1 cartridges (both 95% elution efficiency).

**Figure 2:**
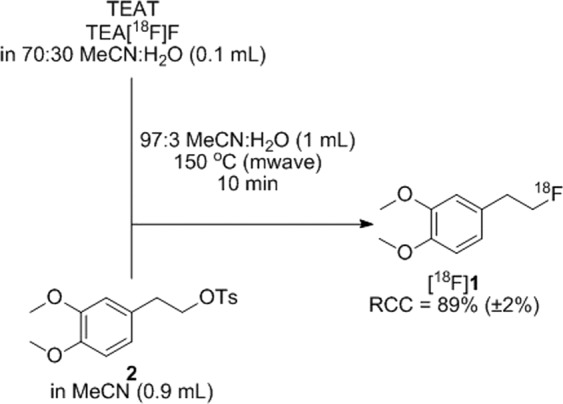
Example radiosynthesis of model compound [^18^F]**1** using the “NAMB” ^18^F-fluorination method (Table 2, entry 11).

The ability of tetraethylammonium salts to facilitate nucleophilic ^18^F-fluorinations in “damp” MeCN or DMSO was assessed using [^18^F]**1** as a model compound (Fig. 2). Cartridge eluates (100 µL) containing 30% water/70% organic solvent or 50% water/50% organic solvent were diluted with a solution of precursor **2** (1.5 mg) in anhydrous organic solvent (900 µL) such that the final reaction volume was 1 mL and the final water content was 3% or 5%. The reaction mixtures were then heated by microwave irradiation (150 °C, 10 min) and assayed by radio-TLC (Table 2). Little difference in RCC was observed when comparing the three AEX reagents or the two organic solvents. However, the RCC of reactions carried out in 97% organic solvent (Table 2, entries 7–12) was generally higher (72–89%) than those carried out in 95% organic solvent (51–72%; entries 1–6). The highest yield was observed using TEATos in 97% MeCN (89%, Fig. 2). See Supplementary Fig. S3 for an example radio-TLC trace of an [^18^F]**1** reaction mixture.

**Table 2:** Reaction conditions evaluated for the ^18^F-labeling of 2.

Entry	AEX Reagent*	Solvent Matrix	% RCC of [^18^F]1 by TLC** (±σ)
1	TEAB	95% MeCN	55 (<1), 56 (1), 59 (<1)
2	TEAB	95% DMSO	56 (1), 62 (<1), 69 (<1)
3	TEAP	95% MeCN	72 (2)
4	TEAP	95% DMSO	65 (<1)
5	TEATos	95% MeCN	53 (1), 59 (<1), 55 (3), 51 (<1)
6	TEATos	95% DMSO	54 (1)
7	TEAB	97% MeCN	88(1)
8	TEAB	97% DMSO	72 (<1)
9	TEAP	97% MeCN	77 (<1)
10	TEAP	97% DMSO	77 (1)
11	TEATos	97% MeCN	89 (2)
12	TEATos	97% DMSO	83 (1)

Reaction conditions: 4.5 µmol (1.5 mg) of **2** in 1 mL, 150 °C, 10 min. *7.8 µmol. Owing to its explosive potential when dry, TEAP is typically sold “damp” (~10% water), which hampers accurate weighing. **Silica gel chromatography with ethyl acetate as mobile phase. Each value is the average of three measurements and represents a unique radiosynthesis.

**Figure 3:**
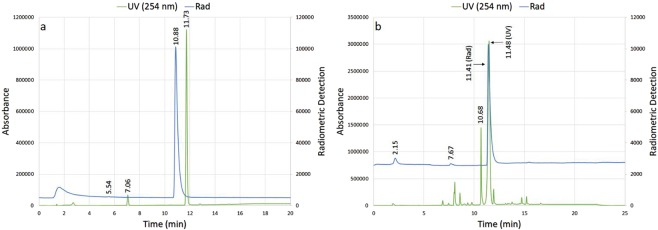
Analytical HPLCs of representative reaction mixtures of (a) model compound [^18^F]**1** and (b) [^18^F]FBA using the “NAMB” strategy. Both traces were obtained using HPLC 1, Program A, as described in the Supplementary Information. (a) Reaction conditions: TEAP, 97% MeCN, 150 °C, 10 min. t_R_ [^18^F]**1** = 10.88 min. t_R_ tosylated precursor **2** = 11.73 min. (b) Reaction conditions: TEAP, 97% DMSO, 150 °C, 10 min. t_R_ [^18^F]**3** = 11.41 min., t_R_ 4 = 11.48 min.

The crude [^18^F]**1** reaction mixtures were largely free of radiochemical byproducts as determined by analytical HPLC (Fig. 3a). Furthermore, as the degree of precursor decomposition has been utilized by others as a metric of overall ^18^F-fluorination reaction “mildness”^9^, it is worth noting that in reaction mixtures using TEAP or TEATos in 97% MeCN, precursor **2** remained largely intact after heating.

**Figure 4:**
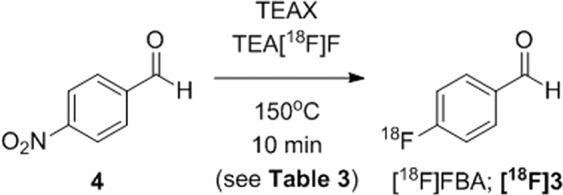
Radiosynthesis of 4-[^18^F]fluorobenzaldehyde ([^18^F]**3**) from 4-nitrobenzaldehyde (**4**). TEAX = TEAB, TEAP or TEATos.

As some previous reports describing non-anhydrous ^18^F-fluorination reactions are limited in either the choice of leaving group (e.g. –OTs only)^11^ or the mechanism of ^18^F incorporation (e.g. S_N_Ar reactions only)^5^, we tested the utility of the “NAMB” approach for the radiosynthesis of the ^18^F prosthetic molecule 4-[^18^F]fluor-obenzaldehyde ([^18^F]FBA; **[**^**18**^**F]3**) from 4-nitrobenzaldehyde (**4**; Fig. 4 and Table 3). Interestingly, this reaction proceeded in non-anhydrous solvent mixtures containing DMSO but not MeCN. As observed for model compound [^18^F]**1**, a decrease in water content from 5% to 3% correlated with an increase in RCC from 43–50% (entries 4–6) to 64–76% (entries 7–9). See Fig. 3b for a representative radio-HPLC trace of the [^18^F]3 reaction mixture and Supplementary Fig. S4 for an example radio-TLC trace.

**Table 3:** Summary of the reaction conditions evaluated for the synthesis of [^18^F]FBA.

Entry	AEX Reagent*	Solvent Matrix	% RCC of [^18^F]FBA by TLC** (±σ)
1	TEAB	95% MeCN	0 (<1)
2	TEAP	95% MeCN	0 (<1)
3	TEATos	95% MeCN	1 (<1)
4	TEAB	95% DMSO	43 (1)
5	TEAP	95% DMSO	45 (1)
6	TEATos	95% DMSO	50 (1)
7	TEAB	97% DMSO	76 (2)
8	TEAP	97% DMSO	68 (3)
9	TEATos	97% DMSO	64 (1)

Reaction conditions: 4-nitrobenzaldehyde 19.9 µmol (3 mg) in 1 mL. *7.8 µmol. Owing to its explosive potential when dry, TEAP is typically sold “damp” (~10% water), which hampers an accurate measurement of TEAP mass. **Silica gel chromatography with ethyl acetate as mobile phase. Values represent an average of three measurements.

**Figure 5:**
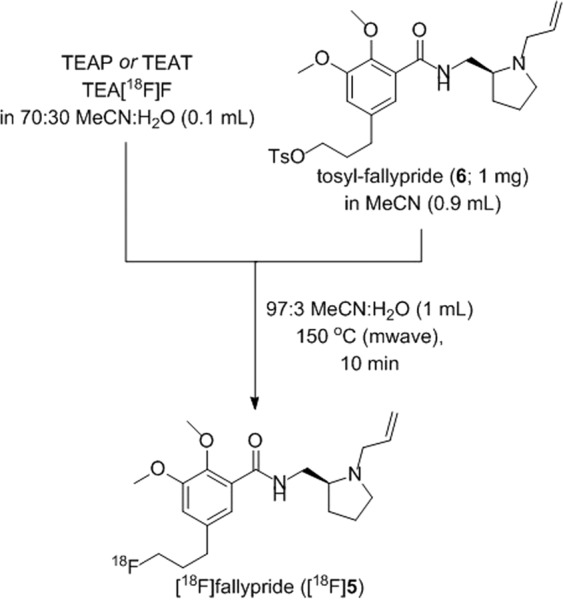
Tetraethylammonium salt-mediated radiosynthesis of [^18^F]fallypride ([^18^F]**5**) under “NAMB” ^18^F-fluorination conditions.

In light of the promising results with [^18^F]**1** and [^18^F]**3**, we sought to utilize “NAMB” ^18^F-fluorination chemistry for the preparation of an established ^18^F-labeled radiopharmaceutical. This method was, therefore, applied to the synthesis of [^18^F]fallypride ([^18^F]**5**; (*S*)-*N*-[(1-allyl-2-pyrrolidinyl)methyl]-5-(3-[^18^F]fluoropropyl)-2,3-dimethoxybenzamide), a clinically useful D_2_/D_3_ receptor antagonist^24,25^ (Fig. 5). The standard synthesis of [^18^F]fallypride is known to be base-sensitive, because of the tendency of the tosyl-fallypride precursor **6** to undergo hydrolysis and elimination side reactions^26^, making this compound a good candidate with which to evaluate this minimally basic synthesis.

For these experiments, [^18^F]F^−^ was eluted from MP-1 columns using TEAP or TEATos (7.8 µmol) in 70% MeCN (100 µL). The eluate was diluted with a solution of **6** (1 mg) in dry MeCN (900 µL), and the reaction mixture was heated by microwave (150 °C, 10 min). See Supplementary Fig. S5 for an example radio-TLC trace. [^18^F]Fallypride was isolated by semi-preparative HPLC followed by solid-phase extraction and formulation in 10% EtOH in saline. Radiolabeling efficiencies (RCCs) of [^18^F]fallypride were generally higher when TEAP was used (64–81%, *n* = 4) vs. TEATos (55–72%, *n* = 5), but this did not translate in a significant difference in the radio-chemical yield (*vide infra*). The amount of precursor used in this reaction (1 mg) was less than that used in other standard [*i.e*. K[^18^F]F-K_2.2.2._/(bi)carbonate]^24,26–28^ and non-standard^4,10,11,22^ syntheses of [^18^F]fallypride (2–40 mg). In contrast to most other [^18^F]fallypride syntheses carried out without azeoptropic drying of the [^18^F]F^−^ ^4,10,11,16^, this reaction was also carried out in a reaction volume (1 mL) that is compatible with typical automated radio-pharmaceutical production systems.

The importance of the interplay between the [^18^F]F^−^ elution conditions and the [^18^F]F^−^ labeling conditions is highlighted by the work of Lemaire *et al*.^6^, who showed that [^18^F]F^−^ could be efficiently stripped from QMA resin with the phosphazine base P_2_Et in “damp” MeCN (0.63% H_2_O). Subsequently, the entire eluate volume (700 µL) was reacted with **6** in dry MeCN (0.5–1 mL) containing 2-*t*-butyl-1,1,3,3-tetramethylguanidine without azeo-tropic drying of the [^18^F]F^−^. However, under these conditions, 20 mg of **6** was required to achieve high RCC (87%), presumably due to the larger final reaction volume (>1 mL) and the presence of a relatively large quantity of a very strong base (P_2_Et; 45 µmol) in the reaction mixture. Investigators were able to achieve an equally high product yield (86%) using only 1 mg of precursor, but this was only possible by employing a small fraction of the total eluate volume (50 μL out of 900 µL, or 5.6% of the total radioactivity), which reduces the amount of P_2_Et present in the reaction mixture from 45 µmol to 2.5 µmol. It is worth noting that P_2_Et does not itself displace the [^18^F]F^−^ from the anion exchange resin. The [^18^F]F^−^ is eluted by an anion, presumably OH^−^, produced *in situ* by P_2_Et deprotonation of the water present in the eluent.

**Figure 6:**
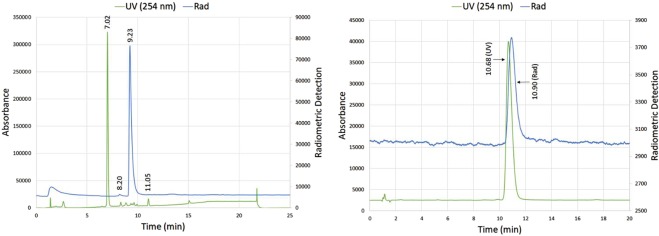
Analytical HPLCs of (a) [^18^F]fallypride ([^18^F]**5**) reaction mixture and (b) co-injection of [^18^F]**5** with non-radioactive standard. (a) HPLC conditions: HPLC 1, Program A, as described in the Supplementary Information. Reaction conditions: TEATos, 97% MeCN, 150 °C, 10 min. [^18^F]**5** t_R_ = 9.23 min. Precursor **6** t_R_ = 11.05 min. (b) HPLC conditions: HPLC 1, Program C (See the Supplementary Information). UV- and γ-detectors were placed in serial, which results in slight differences in t_R_s between [^18^F]**5** (t_R_ = 10.90 min) and [^19^F]**5** (t_R_ = 10.68 min).

The HPLC profiles of [^18^F]fallypride reaction mixtures employing TEAP and TEATos were very similar. In contrast to compound [^18^F]**1**, significant decomposition of the starting material was observed after heating, with the major decomposition product found at 7.02 min in HPLC assays of the crude reaction mixtures (Fig. 6a). Nevertheless, only one small radioactive impurity was consistently observed (Fig. 6a, t_R_ = 8.20 min). Isolated RCYs of [^18^F]**5** using TEAP in three separate experiments were 35, 35 and 42%. Using TEATos, the isolated RCYs in three separate experiments were 31, 32 and 37%. Synthesis times ranged from 78–100 min from start-of-synthesis.

The yield of the NAMB [^18^F]fallypride synthesis is approximately 40% higher than the 25% obtained by Kwon *et al*. in their non-anhydrous synthesis of [^18^F]fallypride^22^. Comparing the yield of the NAMB synthesis to that obtained using standard anhydrous methods, Mukherjee, *et al*. reported yields of 35–42% in their original manual synthesis of [^18^F]fallypride^24^, similar to the results obtained in this study. More recently, other investigators reported yields of 35–40% for the automated synthesis of [^18^F]fallypride^29–31^. The highest yield (68%) was reported by Moon, *et al*. who used 10 µL of a 40% solution of TBAB as the phase transfer catalyst and observed that the yield decreased to less than 50% in the presence of higher amounts of base^26^.

The radiochemical purity of all final products was >99%. Product identity was verified by co-injection of the ^18^F-labeled compound with non-radioactive fallypride (Fig. 6b). Molar activities (MAs) were calculated based on a calibration curve prepared from [^19^F]**5**. The MAs were similar regardless of PTC employed (TEAP: 5, 8 and 13 GBq/µmol; TEATos: 4 and 7 GBq/µmol). The moderate MA is attributable to our [^18^F]F^−^ source (*i.e*. [^18^F]F^−^ flushed from transfer lines after clinical production of other tracers) and to the relatively small amounts of [^18^F]F^−^ employed in the syntheses (74–370 MBq). Higher MAs can be achieved using larger quantities of freshly prepared [^18^F]F^−^.

Overall, for the synthesis of [^18^F]fallypride under NAMB conditions, we did not observe a significant advantage of TEATos over TEAP in terms of ease of use (e.g. solubility), precursor tolerance (*i.e*. reaction “mildness”), radiochemical yield, or molar activity.

## Conclusions

Solutions of tetraethylammonium tosylate and tetraethylammonium perchlorate in non-anhydrous solvent mixtures offer a straightforward means to efficiently extract [^18^F]F^−^ from small AEX columns and facilitate the synthesis of ^18^F-labeled aromatic and aliphatic compounds without the need for the azeotropic drying step that is ubiquitous in ^18^F-PET chemistry. Since these anions do not contribute to the basicity of the reaction mixture, we describe this approach as “non-anhydrous, minimally basic” (“NAMB”) ^18^F-fluorination chemistry. Tetraethylammonium tosylate offers a particularly attractive alternative to standard reagents because the tosylate anion is both non-basic and non-oxidizing and because it is already present in many nucleophilic ^18^F-fluorination reactions as a leaving group.

As shown by the synthesis of [^18^F]fallypride, “NAMB” labeling conditions can be used to prepare ^18^F-PET tracers from commercially available, GMP-compliant precursor molecules and single portions of [^18^F]F^−^ without the need to dry the [^18^F]F^−^ prior to use. Furthermore, the “NAMB” method can accommodate volumes of aqueous [^18^F]F^−^ (1–2 mL) and concentrations of precursor (1–3 mg in 1 mL) that are commonly used in automated synthesis systems. Further improvements in this technique are anticipated as “NAMB” chemistry is evaluated for the synthesis of a wider variety of clinically relevant ^18^F radiopharmaceuticals.

## Experimental Section

### Example radiosynthesis of [^18^F]fallypride

#### *Preparation of active [*^18^F]fluoride

An aliquot of [^18^F]F^−^ (90 MBq, 2.44 mCi) in [^18^O]H_2_O was diluted to 1.5 mL with H_2_O and the [^18^F]F^−^ was trapped on an MP-1 anion-exchange column (MedChem Imaging, carbonate form, 10–12 mg), which was previously activated with H_2_O (1 mL). After washing the column with anhydrous MeCN (3 mL), Ar was passed through the column for 10 min. Fluorine-18 was eluted from the column in the reverse direction into a microwavable test tube using a solution of tetraethylammonium tosylate (TEATos, 23.5 mg/mL, 100 µL) in 7:3 MeCN:H_2_O. Residual liquid was removed from the column using a syringe filled with air (10 mL).

#### ^18^*F-labeling reaction*

Tosyl-fallypride (**6**; 1 mg) in dry MeCN (900 µL) was added to the [^18^F]F^−^ solution and the tube was crimp-sealed, magnetically stirred for 20 sec, and heated (microwave) to 150 °C for 10 min. After removing small aliquots for silica gel radio-TLC (10% MeOH in CH_2_Cl_2_, 72 ± 2% RCC, *n* = 4) and analytical HPLC (HPLC 1, Program A in the Supplementary Information), the reaction mixture was diluted with 0.1% TFA in water (1 mL) and injected onto a semi-preparative HPLC column (HPLC 2, Program B). The product was collected, diluted with water (50 mL), and trapped on a Sep-Pak® C_18_ Light cartridge that was previously activated with EtOH (3 mL) and water (10 mL). After washing the column with water (5 mL), [^18^F]fallypride was eluted with EtOH (1 mL) and diluted with 0.9% saline (9 mL). The final formulation was passed through a 0.2 micron filter to afford 22.1 MBq (596 µCi) of [^18^F]fallypride (21% non-decay corrected, 37% decay corrected). Product identity and molar activity were assessed by HPLC (HPLC 1, Program C). The synthesis time was 88 min. from start-of-synthesis.

## Supplementary information

Supplementary Information.

## Data Availability

All data reported in this manuscript are available upon reasonable request by contacting the corresponding author.
