# Supplementary material for: A non-anhydrous, minimally basic protocol for the simplification of nucleophilic 18F-fluorination chemistry
Source: Sci Rep. 2020 Apr 22;10:6818. doi: 10.1038/s41598-020-61845-y (PMC7176689; doi:10.1038/s41598-020-61845-y)
Supplement: Supplementary file 1 — Supplementary Information. [file 41598_2020_61845_MOESM1_ESM.docx]

**Supporting Information**

**A non-anhydrous, minimally basic protocol for the simplification of nucleophilic ^18^F-fluorination chemistry**

J. A. H. Inkster, V. Akurathi, A. W. Sromek, Y. Chen, J. L. Neumeyer and A. B. Packard

**Table of Contents**

General Information 2

Non-radioactive syntheses related to model compound [^18^F]**1** (Figure S1). 3

Photograph of small MP-1 cartridge fitted with Luer-lock/hose barb adapter (Figure S2) 4

Representative radio-TLC traces (Figures S3, S4 and S5) 5

NMR spectra 7

References 11

**General Information**

*Chemicals*

Most reagents and solvents were purchased from Acros Organics (Fair Lawn, NJ), Alfa Aesar (Ward Hill, MA), Fisher Scientific (Hampton, NH), Oakwood Chemical (Estill, SC), or Millipore-Sigma (St. Louis, MO) and were used without further purification. Tosyl-fallypride was obtained from ABX, GmbH. (Radeberg, Germany). Silica gel (40-63 µm) for flash chromatography was obtained from Silicycle (Quebec City, Canada). MP-1 and QMA anion-exchange cartridges (both carbonate form) were obtained from MedChem Imaging (Boston, USA). Sep-Pak® C18 and tC18 solid-phase extraction cartridges were obtained from Waters (Milford, MA).

*Thin Layer Chromatography (TLC)*

TLC was performed on pre-coated silica gel 60 F_254_ aluminum sheets from EMD Millipore (Billerica, MA). Non-radioactive compounds were visualized under ultraviolet light at 254 nm. A Fujifilm BAS-5000 phosphor imager with Multi Gauge v3.0 software was used to visualize ^18^F on the TLC plates.

*Liquid Chromatography*

The HPLC systems described below, including data acquisition modules, are Shimadzu Prominence brand. Gamma detectors were optimized for 511 keV photons.

**HPLC 1.** Analytical. Pump: LC-20AD. Diode array detector (DAD): SPD-M20A. Radiation detection: Harshaw NaI(Tl) detector with Canberra NIM electronics.

**HPLC 2.** For manual preparations. Pump: LC-20AT. UV/Vis detector: SPD-20A. Radiation detection: Carroll & Ramsey Model 105S.

**Program A.** Column: EMD Millipore Purosphere® RP-18 endcapped, 5 µm, 4 mm × 125 mm. Solvent system: gradient elution, 10% MeCN in H_2_O containing 0.1% trifluoroacetic acid (TFA) for 2 min, raised to 90% MeCN in H_2_O containing 0.1 % TFA over 13 min, hold for 5 min, flow rate = 1 mL/min. Detector: 190-800 nm (DAD).

**Program B.** Column: ES Industries Chromegabond WR C18, 5 µm, 120 Å, 9.6 mm × 250 mm. Solvent system: gradient elution, 10% MeCN in H_2_O containing 0.1% TFA for 2 min, raised to 90% MeCN in H_2_O containing 0.1 % TFA over 13 min, hold for 5 min, flow rate = 3 mL/min. Detector: 254 nm.

**Program C.** Column: EMD Millipore Purosphere® RP-18 endcapped, 5 µm, 4 mm × 125 mm. Solvent system: isocratic elution, 25:75 MeCN:H_2_O containing 0.1% TFA, flow rate = 1 mL/min. Detector: 190-800 nm (DAD).

*NMR*

^1^H and ^13^C NMR spectra were recorded with either a Varian 400-MR or a Varian Mercury 300 spectrometer (Palo Alto, CA). NMR solvents were obtained from Cambridge Isotope Laboratories (Andover, MA). Chemical shifts (δ) are reported in ppm relative to the hydrogenated residue of the deuterated solvents.

*Elemental Microanalysis (EA)*

EA was carried out by Atlantic Microlab, Inc. (Norcross, GA).

*Microwave*

Microwave heating of radiolabeling reactions were carried out on a Biotage Initiator+ single-mode device with an infrared pyrometer and a pressure control system. The reactions were carried out using crimped glass reaction vessels (0.5-2 mL size) containing magnetic stir vanes.

*Production of [^18^F]fluoride ion.*

No-carrier-added [^18^F]fluoride was produced by proton bombardment of 3.5 mL of [^18^O]water on a GE 16.5 MeV PETtrace cyclotron at the Brigham and Woman's Hospital BICOR facility (Boston, MA). Approx. 96 GBq [^18^F]F^-^ was obtained in this fashion, which was used for purposes unrelated to this study. The transfer lines were then flushed, which afforded an additional 740–1850 MBq for use in our experiments. For each individual experiment, a fraction of the radioactivity was diluted with ultrapure water to 1-2 mL prior to use. For methodology experiments, 10-580 MBq [^18^F]F^-^ was employed, while 74-370 MBq were used for [^18^F]fallypride syntheses.

**Non-radioactive syntheses related to model compound [^18^F]1**

**Figure SI.** Synthesis of model compound 1 and precursor 2.

*4-(2-fluoroethyl)-1,2-dimethoxybenzene (****1****).*

Compound **1** was prepared as previously described.[^1^](#_ENREF_1) Yield: 57% (105 mg).

^1^H NMR (400 MHz, CDCl_3_) δ 2.95 (dt, *J* = 23.3, 6.5 Hz, 2H), 3.85 (s, 3H), 3.87 (s, 3H), 4.60 (dt, *J* = 47.5, 6.7 Hz, 2H), 6.82 (s, 1H), 6.79 – 6.72 (m, 2H).

^13^C NMR (101 MHz, CDCl_3_) δ 36.40 (d, *J* = 20.2 Hz), 55.73, 55.80, 84.17 (d, *J* = 168.9 Hz), 111.25, 112.16, 120.82, 129.59 (d, *J* = 6.4 Hz), 147.75, 148.86.

^19^F NMR (376 MHz, CD_2_Cl_2_) δ -221.35 (tt, *J* = 48.4, 29.0 Hz).

Elemental Anal. C_10_H_13_FO_2_. Calcd. C 65.20, H 7.11. Found C 64.92, H 7.24.

*3,4-Dimethoxyphenethyl 4-methylbenzenesulfonate (****2****).*

Compound **2** was prepared as previously described.[^2^](#_ENREF_2) Yield: 88% (3.24 g).

^1^H-NMR (400 MHz, CD_2_Cl_2_) δ 2.42 (s, 3H), 2.86 (t, *J* = 6.8 Hz, 2H), 3.74 (s, 3H), 3.79 (s, 3H), 4.17 (t, *J* = 6.8 Hz, 2H), 6.58 (d, *J* = 1.9 Hz, 1H), 6.64 (dd, *J* = 8.2, 2.0 Hz, 1H), 6.75 (d, *J* = 8.1 Hz, 1H), 7.29 (d, *J* = 8.0 Hz, 2H), 7.64 (d, *J* = 8.3 Hz, 2H).

^13^C-NMR (100 MHz, CD_2_Cl_2_) δ 21.27, 34.77, 55.58, 55.73, 70.93, 111.50, 112.23, 120.86, 127.66, 128.89, 129.69, 132.92, 144.84, 148.17, 149.11.

Elemental Anal. C_17_H_20_O_5_S. Calcd. C 60.70 H 5.99. Found C 60.78 H 6.08.


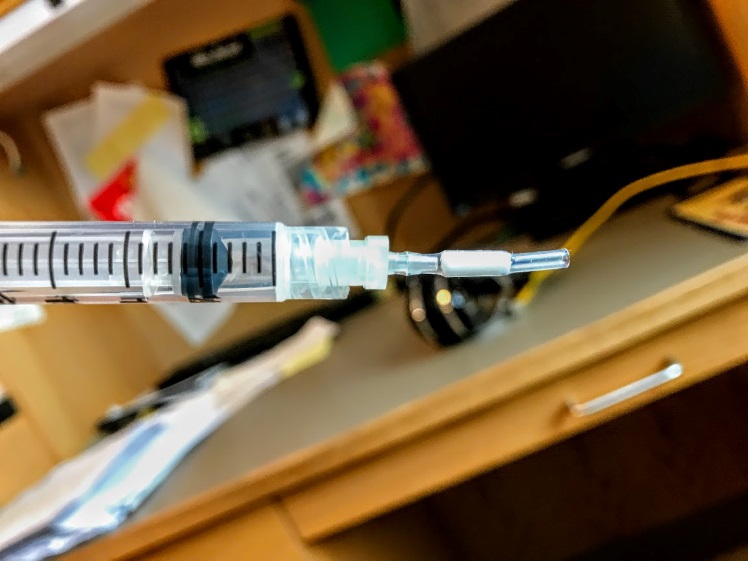


**Figure S2.** A small MP-1 cartridge connected via Luer-lock/hose barb adapter to a 3 mL syringe.


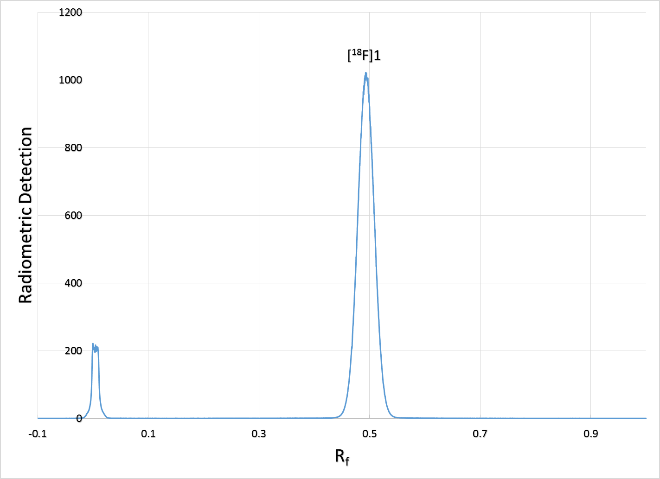


**Figure S3.** Example radio-TLC of [^18^F]**1** (TEAT, 97% MeCN, 150 °C, 10 min). RCC = 92%. Eluent = ethyl acetate.


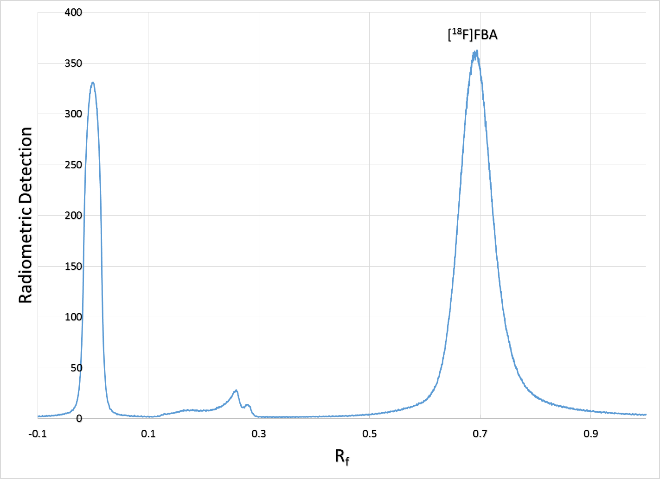


**Figure S4.** Example radio-TLC of 4-[^18^F]fluorobenzaldehyde (**[^18^F]FBA**, TEAP, 97% DMSO, 150 °C, 10 min). RCC = 71%. Eluent = ethyl acetate.

**
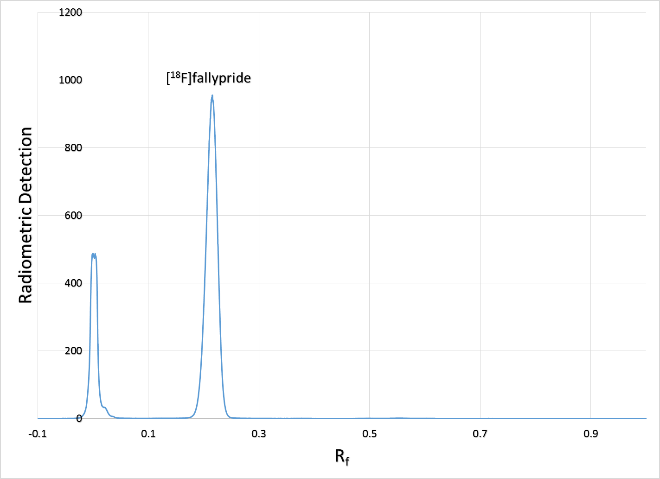
**

**Figure S5.** Example radio-TLC of [^18^F]fallypride (TEAT, 97% MeCN, 150 °C, 10 min). RCC = 75%. Eluent = 10% MeOH in CH_2_Cl_2_.

**NMR spectra**

Compound **1.** ^1^H NMR.

Compound **1.** ^13^C NMR.

Compound **1**. ^19^F NMR.

Compound **2**. ^1^H NMR.


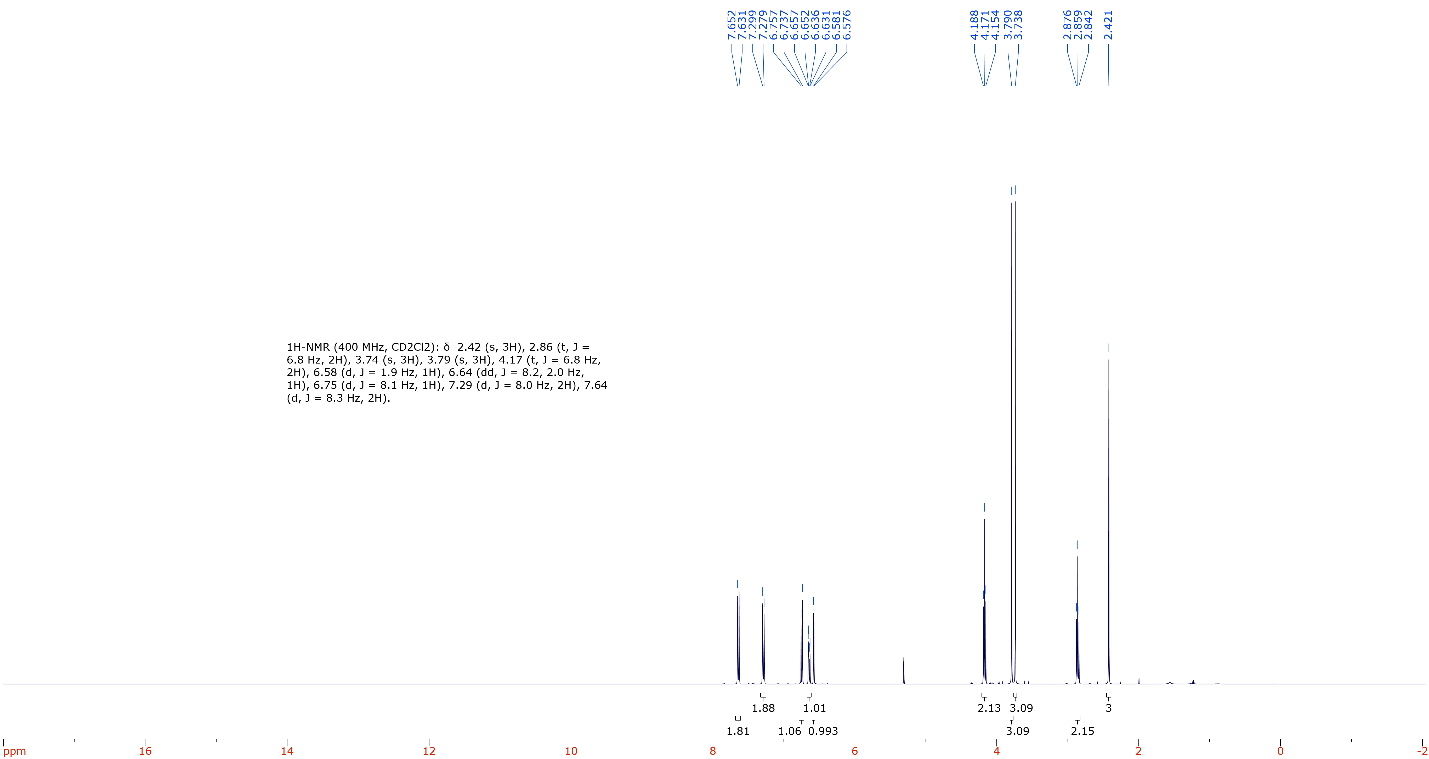


Compound **2.** ^13^C NMR.


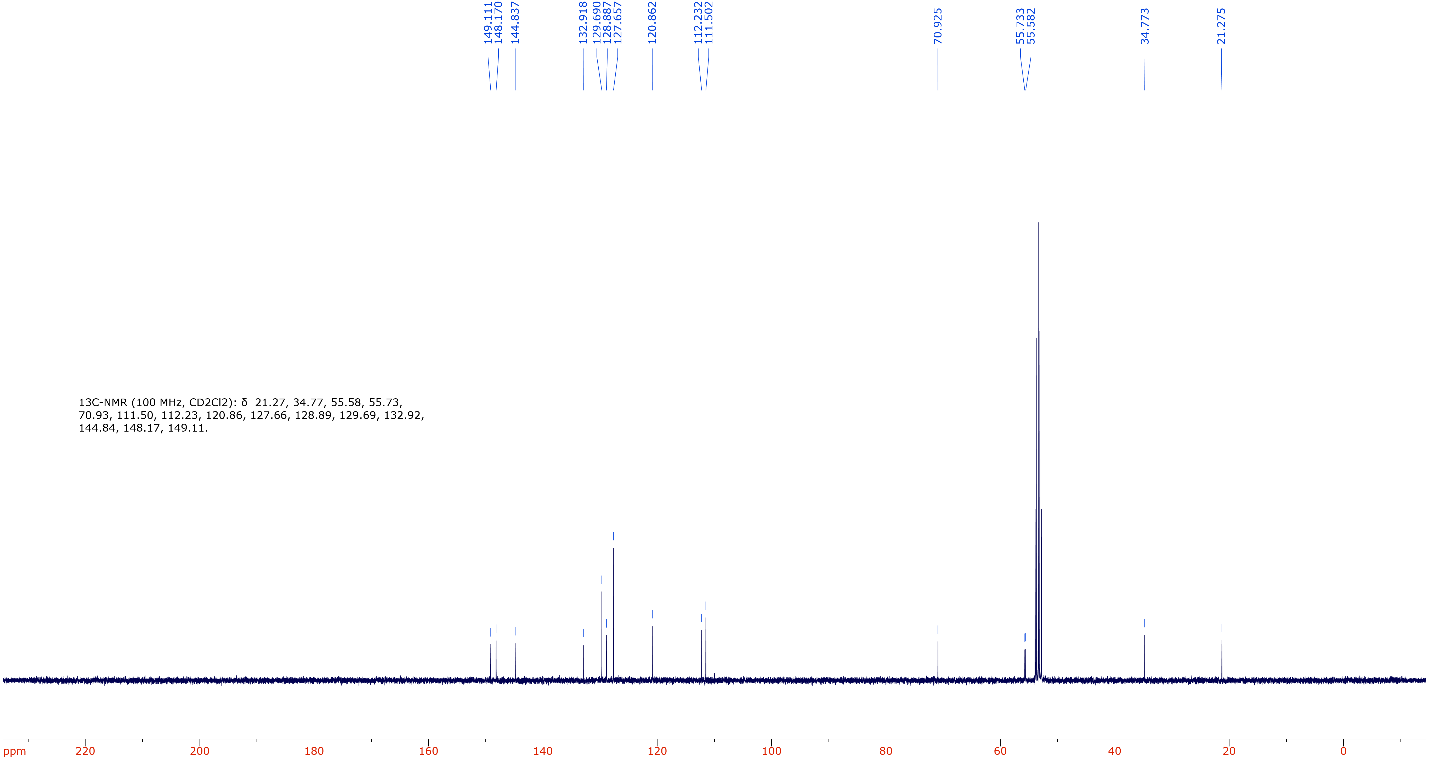


# References

1 Swenton, J. S., Bonke, B. R., Chen, C. P. & Chou, C. T. Anodic-oxidation studies of para-methoxyanilides - A general method for preparation of acylated quinone imine ketals. *J Org Chem* **54**, 51-58, (1989).

2 Shaw, M. H., Croft, R. A., Whittingham, W. G. & Bower, J. F. Modular access to substituted azocanes via a rhodium-catalyzed cycloaddition-fragmentation strategy. *J Am Chem Soc* **137**, 8054-8057, (2015).
